# A machine learning system to identify progress level of dry rot disease in potato tuber based on digital thermal image processing

**DOI:** 10.1038/s41598-023-50948-x

**Published:** 2024-01-23

**Authors:** Saeid Farokhzad, Asad Modaress Motlagh, Parviz Ahmadi Moghaddam, Saeid Jalali Honarmand, Kamran Kheiralipour

**Affiliations:** 1 Department of Mechanical Biosystems, Faculty of Agriculture, Urmia University, Urmia, Iran; 2 Department of Agronomy and Plant Breeding, Campus of Agriculture and Natural Resources, Razi University, Kermanshah, Iran; 3 Mechanical Engineering of Biosystems Department, Faculty of Agriculture, Ilam University, Ilam, Iran

**Keywords:** Microbiology, Plant sciences

## Abstract

This study proposed a quick and reliable thermography-based method for detection of healthy potato tubers from those with dry rot disease and also determination of the level of disease development. The dry rot development inside potato tubers was classified based on the Wiersema Criteria, grade 0 to 3. The tubers were heated at 60 and 90 °C, and then thermal images were taken 10, 25, 40, and 70 s after heating. The surface temperature of the tubers was measured to select the best treatment for thermography, and the treatment with the highest thermal difference in each class was selected. The results of variance analysis of tuber surface temperature showed that tuber surface temperature was significantly different due to the severity of disease development inside the tuber. Total of 25 thermal images were prepared for each class, and then Otsu’s threshold method was employed to remove the background. Their histograms were extracted from the red, green, and blue surfaces, and, finally, six features were extracted from each histogram. Moreover, the co-occurrence matrix was extracted at four angles from the gray level images and five features were extracted from each co-occurrence matrix. Totally, each thermograph was described by 38 features. These features were used to implement the artificial neural networks and the support vector machine in order to classify and diagnose the severity of the disease. The results showed that the sensitivity of the models in the diagnosis of healthy tubers was 96 and 100%, respectively. The overall accuracy of the models in detecting the severity of tuber tissue destruction was 93 and 97%, respectively. The proposed methodology as an accurate, nondestructive, fast, and applicable system reduces the potato loss by rapid detection of the disease of the tubers.

## Introduction

Global human population growth necessitates of the mass supply of foods. Fluctuations in food prices in international markets due to lack of production, diseases, distribution and so on cause food shortages and social unrest. In this regard, agriculture and food processing sectors must be evaluated to move in sustainable production path^1^. So, it is necessary to make a progress in food production and focus on the production of more nutrient foods, such as the potato, to reduce this risk^2^. However, limitations and expensiveness of preparing and maintaining seed tubers, potato rot in icehouses or warehouses, and the development of potato diseases during the storage period are among the major problems of potato farmers^3^. Potato tuber or seed rot is among the most important and damaging diseases of potatoes after harvesting, storing, and planting^4^, the most prevalent of which is dry rot caused by some species of Fusarium, especially *Fusarium solani* which is highly pathogenic^5^. Potato dry rot reduces the germination power of seeds, rots the tubers, develops contamination, and finally increases crop loss^6^. It is hence necessary to detect and discard rotted potatoes to prevent this disease and reduce mortalities. Also, identifying the progress level of the disease level can help in early detection of that to find a solution for decreasing potato loses.

In conventional methods of detecting rotted potato tubers, which are destructive and time-consuming, potato tubers should be examined one by one and the damaged specimens should be isolated^7^. To increase crop yield and the quality of potato storage, it is necessary to propose quick and accurate methods for diagnosing potato dry rot and infections in potatoes and also identify the progress level of the disease. In these regard, processing of images captured by different electromagnetic bands has vast applications to detect contamination in agricultural and food products^8,9^. One of these bands is infrared domain that is acquired by thermography systems^10,11^.

Thermography is a powerful technique to identify uniformity and defect in products via receiving thermal radiations and also detects such defects which cannot be identified by visible images^12,13^. Also, it is non-destructive approach that has recently attracted great attention as a useful tool for food quality and safety assessment^14,15^. The advantages of this method caused to apply thermal imaging technique to detect diseases in plant leaves^16–20^ and diagnosis of fungal contamination in crops^21–23^.

Besides detecting of fungal infection, predicting the level of fungal growth in agricultural and food products is important in postharvest management systems to find a solution and so decrease the product losses^24,25^. In case of potato, the disease spreads quickly in the stores and in future developing stages it destroys the internal tissue of the potato tuber without any disease symptoms in the surface of the tubers so that it cannot be detected by visible inspections.

Machine learning by applying statistical and artificial methods is used to predict and classify the data extracted from different images^12,13^. Among them, artificial neural network and support vector machine are mainly used in classification of images^26,27^. So the aim of the present study is to employ digital thermal imaging coupled with machine learning to diagnose the potato dry rot in different disease progress levels. The novelty of the present research is predicting the severity of tuber’s internal texture by detecting dry rot level using digital thermal imaging.

## Results

### Emissivity coefficient of tubers

The results of the present research showed that the emissivity coefficient of potato tubers was 0.89 ± 0.1. Almeida et al.^28^ reported an emissivity coefficient of 0.8 for potato tubers. The different results of these two studies can be attributed to the difference in potato cultivars.

### Thermal images

**Figure 1:**
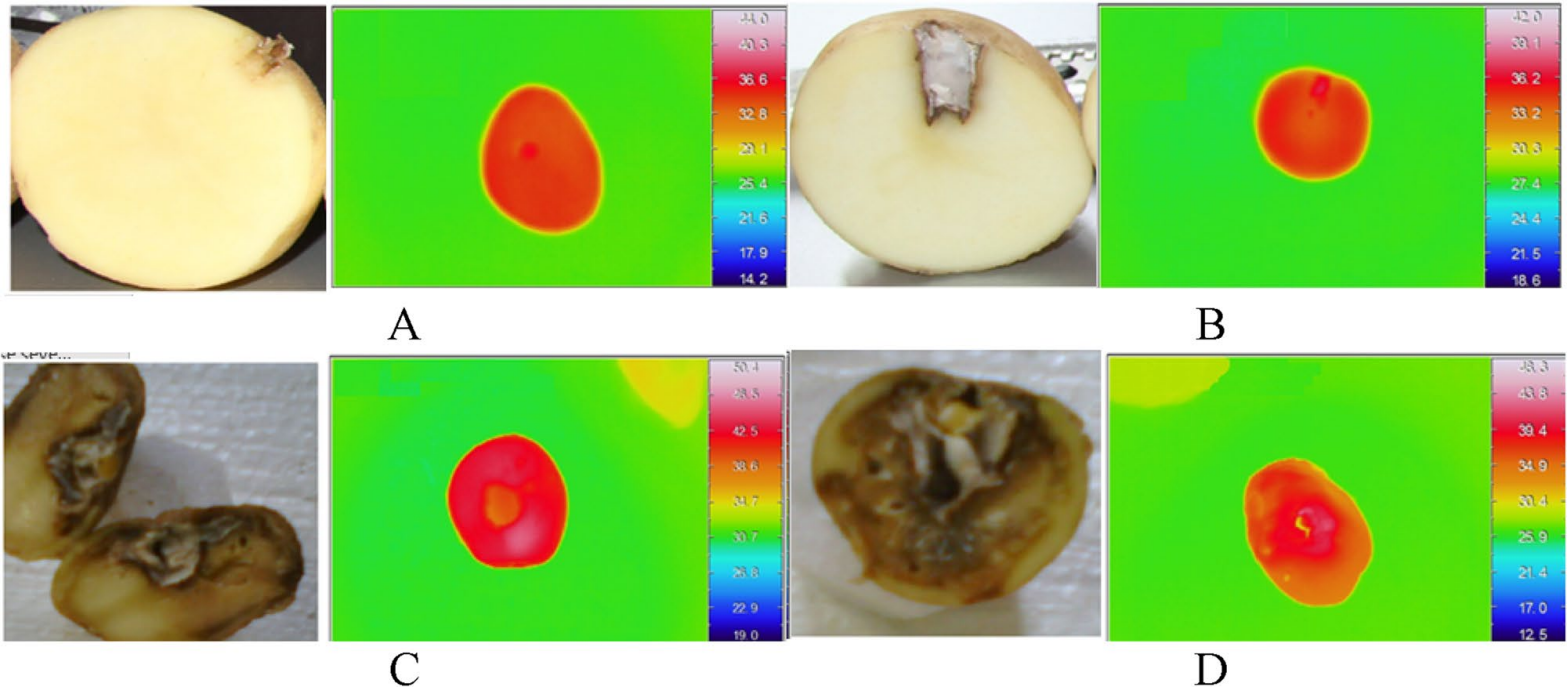
The visible and thermal images to show severity of tuber destruction based on the Wiersema Criteria. (**A**) Healthy tuber, (**B**) first-degree, (**C**) second-degree, and (**D**) third-degree destruction.

After acquiring the thermal images, the internal tissue of the potato tubers was destructively inspected. Figure 1 shows the thermal images of some tubers and visible images of the internal tissues of those. In the development of the Grade A disease, there were no signs of disease growth in the internal tissue, and there were no signs of uneven temperature distribution on the surface of the tuber in the thermal image. With the development of the disease, a part of the inner tissue of the tuber was destroyed, and the symptoms of this degradation have shown themselves in the thermal image as an area with non-uniform temperature distribution. Similarly, with the development of the disease and the destruction of the internal tissue of the tuber, the changes in thermal images were observed more intensely.

Generally, the results showed that in the absence of dry rot disease inside the tuber, its thermal image has a uniform temperature distribution and no heterogeneity was observed in the image tissue. With the onset of the disease inside the tuber and the destruction of its internal tissue, the thermal images also underwent changes, so the results proved that the development of dry caries in the tubers can be detected non-destructively by thermal imaging technique.

### Analysis of variance of tuber surface temperature

The results of the analysis of variance about the effects of development (severity of destruction) of potato dry rot on the surface temperature of tubers indicated that tuber destruction degree (severity of disease), heater temperature, cooling duration, and interaction of treatments affected the surface temperature of tubers (*p* ≤ 0.01) (Table 1). As mentioned earlier, the tubers were heated by an oven for 60 s and part of the absorbed heat penetrated into tubers; if the tuber texture was homogenous, the heat would uniformly penetrate into tubers. On the other hand, the porosity of the tuber texture increases as it is further destructed; the greater the degree of tuber destruction, the greater the tuber hollowness and porosity.

**Table 1:** The effects of the studied factors on the tuber surface temperature.

Sources of variations	Degree of freedom	Sum of squares	Mean of squares	F value
R^#^	24	0.54	0.02	1.42^ns^
A	3	811.11	270.37	17,058.93**
B	1	14,547.57	14,547.57	917,871.47**
A × B	3	24.69	8.23	519.33**
C	3	997.04	332.35	20,696.27**
A × C	9	23.72	2.64	166.65**
B × C	3	441.69	147.23	9189.40**
A × B × C	9	6.95	0.77	48.71**
Error	744	11.79	0.02	
Coefficient of variations				7.11%

^#^R is the replications, A is the degree of tuber destruction, B is the heater temperature, and C is the cooling time.

**and ^ns^ are significant at 0.05 level and non-significant, respectively.

Therefore, the heat penetration rate in tubers with destructed texture is lower than in healthy tubers. In other words, there is a difference between destructed tubers and healthy ones in terms of heat penetration. Moreover, there is a difference between tubers with varying degrees of texture destruction in the post-heating rate of cooling. It can be hence concluded that any spoilage or damage to the potato tuber texture can change their heating and cooling rates. On the other hand, the degree of tuber destruction can be determined and classified more accurately if there is a greater difference between the tuber surface temperature and degrees of texture destruction. It was hence necessary to select a treatment with the highest thermal difference between different degrees of tuber texture destruction. To this end, the mean interactive effects of heater temperature and cooling time was analyzed using Duncan's multiple range test (MRT). The mean tuber surface temperature for different degrees of tuber texture destruction was compared in pairs. The results showed that the greatest difference between the mean tuber surface temperatures was observed in the treatment of heating at 90 °C and cooling time in 70 s, which was selected as the best treatment to achieve the highest thermal difference between different degrees of tuber texture destruction (Table 2). Based on the results, the thermographs obtained from the treatment of heating at 90 °C and cooling time of 70 s were used to classify the degree of tuber texture destruction by using ANN and SVM.

**Table 2:** Comparison of the tuber mean temperature difference between different degrees of tuber texture destruction.

Heating temperature (°C)	Cooling time (s)	Temperature difference between the various stages of tuber texture destruction		
		Healthy-G1^#^	G1- G2	G2- G3
60	10	0.45f.^##^	0.59^ cd^	0.73^e^
	25	0.68^e^	0.78^b^	0.90^d^
	40	0.63^e^	0.57^d^	1.02^c^
	70	0.93^c^	0.70^bc^	1.09^c^
	Mean	0.67	0.65	0.94
90	10	0.83^d^	0.74^b^	1.19^b^
	25	1.01^c^	0.75^b^	1.01^c^
	40	1.28^b^	0.82^b^	0.92^d^
	70	**1.40**^**a**^	**1.34**^**a**^	**1.54**^**a**^
	Mean	1.13	0.91	1.17

^#^G1 is the disease development grade 1, G2 is the disease development grade 2, G3 is the disease development grade 3.

^##^Non similar letters in each column indicates significant difference at 5% probability level.

Significant values are in [bold].

### Detection of the severity of tuber texture destruction

This section presents the results related to the classification of tuber texture destruction based on ANN and SVM. Tables 3 and 4 show the classification result as a confusion matrix for ANN and SVM, respectively. The performance of ANN and SVM for all potato groups was evaluated based on the confusion matrix. The sensitivity of ANN in distinguishing healthy tubers from others as well as firs-, second-, and third-degree tuber texture destruction was obtained as 96, 88, 92, and 96%, respectively. The overall accuracy of ANN and SVM in determining the severity of tuber texture destruction were 93 and 97%, respectively. In addition, the sensitivity of SVM in distinguishing healthy tubers from others as well as firs-, second-, and third-degree tuber texture destruction were equal to 100, 92, 96, and 100%, respectively. The results indicated that SVM can detect dry rot in potato tubers with an accuracy of 100% but its mean accuracy in detecting the disease severity was 96%.

**Table 3:** The confusion matrix obtained from the ANN method.

	Healthy	G1	G2	G3	Accuracy (%)
Healthy	24	1	0	0	96
G1^#^	0	22	3	0	88
G2	0	1	23	1	92
G3	0	0	1	24	96
	Total Accuracy of ANN, 93%				

^#^G1 is the disease development grade 1, G2 is the disease development grade 2, G3 is the disease development grade 3.

**Table 4:** The confusion matrix obtained from the SVM method.

	Healthy	G1	G2	G3	Accuracy (%)
Healthy	25	0	0	0	100
G1^#^	0	23	2	0	92
G2	0	1	24	0	96
G3	0	0	0	25	100
	Total Accuracy of SVM, 97%				

^#^G1 is the disease development grade 1, G2 is the disease development grade 2, G3 is the disease development grade 3.

## Discussion

This study aimed to apply active thermography in order to detect the severity of tuber texture destruction caused by dry rot. The analysis of variance results about the effects of potato dry rot on the tuber surface temperature showed that the severity of tuber texture destruction affected the tuber surface temperature. The best treatment for thermography that produced the greatest difference between healthy and dry rotted tubers in the surface temperature was the treatment of heating at 90 °C and cooling in 70 s. The results also demonstrated that the sensitivity of ANN and SVM in distinguishing healthy tubers from others was 96 and 100%, respectively. In addition, the overall accuracy of ANN and SVM in determining the severity of tuber texture destruction were 93 and 97%, respectively. This result showed the higher ability of SVM in identifying the disease in potato.

Many researchers have proven the efficiency of active thermography in non-destructive spoilages and damages as well as evaluation of crops and food products. The accuracy of the present research is comparable with the previous research with accuracy of 86.3–99% for detecting different stages of fungal infection in pistachio kernels^29^.

The high accuracy of SVM classifier model in the present study can be attributed to thermography under controlled conditions inside the black box. The extraction of 38 features from different levels of the images and co-occurrence matrix made it possible to track any thermography change to one of the features, adding to the accuracy and precision of classification model. So this methodology as a non-destructive system can assist the farmers via reduces the potato loss by detecting the disease because the internal disease cannot be detected by visual inspection and if the presence of the disease is not detected, it spreads quickly in the stores and destroys other stored tubers.

Based on the results, it can be concluded that the developed thermography systems in the present research can detect internal disease in potato tubers with high accuracy and hence assist to decreases the crop and economic loss. So the methodology as a fast and non-destructive system can assist the farmers via reduces the potato loss by detecting the disease. To increase the accuracy of disease detection and identifying the progress level of that in potato, other features such as extracted data based on the fast furrier transform (FFT) data and novel classification methods such as convolutional neural networks can be applied in future researches.

## Methods

### Potato samples

In the present study, the potato samples were from the Diamant variety because the variety has the highest susceptibility to the dry rot disease. The required potato samples were selected from the potato storage silos in Razan, Hamadan, Iran. All potato samples had completely healthy appearance. The tubers were transported from the storage systems after storing for 3 months at 4–6 °C.

### Inoculation of samples

**Figure 2:**
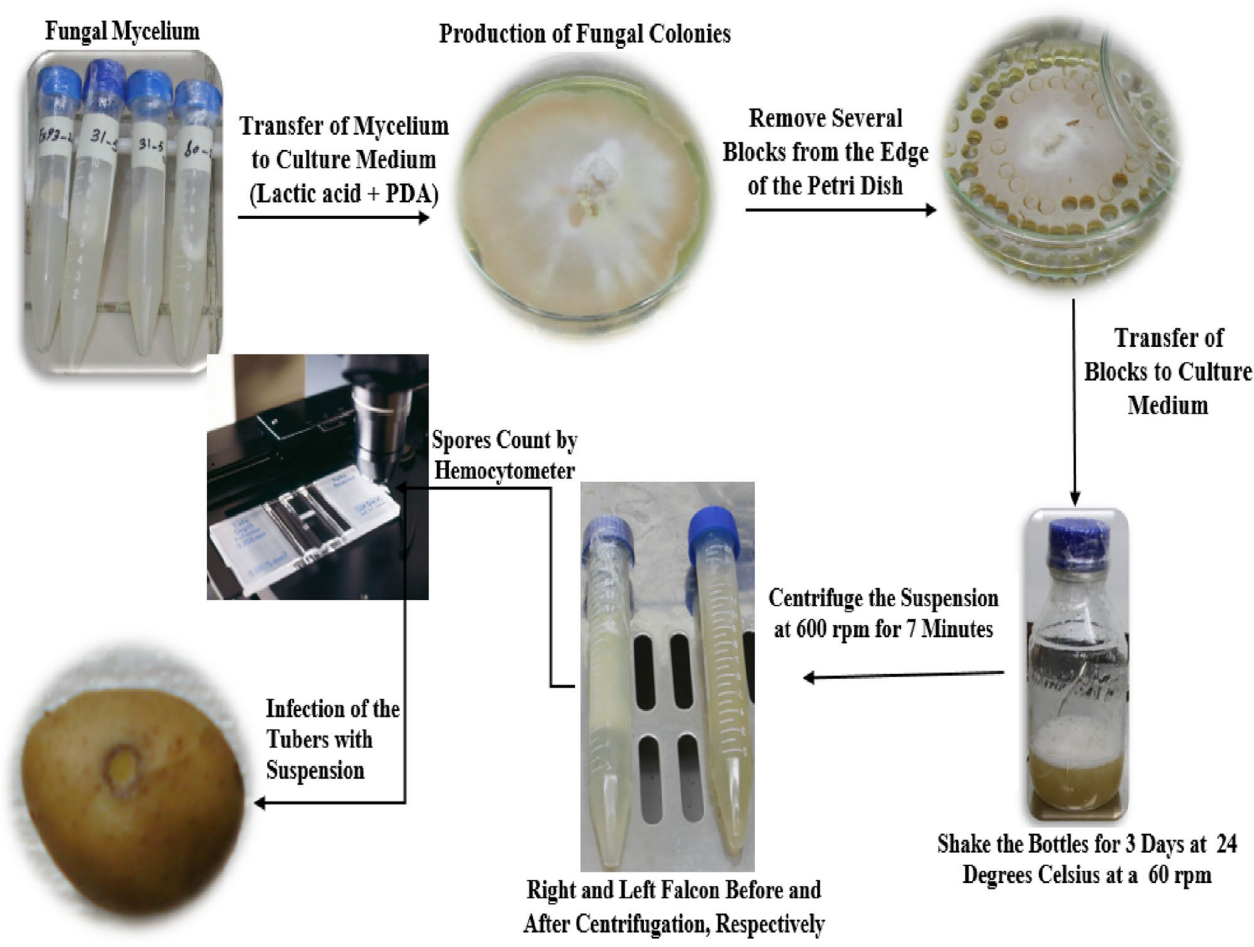
Infection of the potato tubers.

The conducting steps to infect the potato tubers have been depicted in Fig. 2. Mycelium of *Fusarium solani* was obtained from the Department of Plant Protection of Urmia University, Urmia, Iran, and was added to a medium containing lactic acid and PDA in a biological cabinet under sterile conditions. The petri dishes were then kept in an incubator at 25 °C for one week. After fungal colonies and abundant spore were produced, some blocks were removed from the sides of petri dishes and transferred to a liquid culture medium (potato extract + distilled water). The suspensions were then shaken at 60 rpm at 24 °C for 3 d. Spore suspensions were centrifuged at 6000 rpm for 7 min to obtain smooth and clear suspension^30^. After centrifuging the suspensions, the concentration of suspensions was measured by a hemocytometer. The concentration for inoculating the samples was 10^4^ spores per ml^32^. A high speed digital centrifuge, model HS 18,500 R, Farzaneh Arman Co., Iran was used. The maximum rotational speed of the machine is 18,500 rpm and Its Max. RCF is equal to 23,797 g.

One hundred healthy potato tubers were selected and artificially inoculated with the pathogen of potato dry rot. To this end, an incision of 5 mm was made on the tuber to inject 0.4 ml of the suspension containing 10^4^ *F. solani* spores per ml into the tuber texture and then the incision was covered with solid paraffin^31,32^. The control potato samples were inoculated with sterile distilled water. The storage condition of healthy samples was the same as those of spore infected samples but the healthy samples were inoculated with distilled water. In total, 75 and 25 potato tubers were inoculated with the pathogen and distilled water, respectively. Then the tubers were kept in an incubator in the dark environment at 25 °C to provide enough opportunity and the necessary environmental conditions for the pathogen to cause disease^33^. After the tubers were fully inoculated, 25 tubers per week were removed from the incubator for preparing thermographs.

### Thermal imaging system

**Figure 3:**
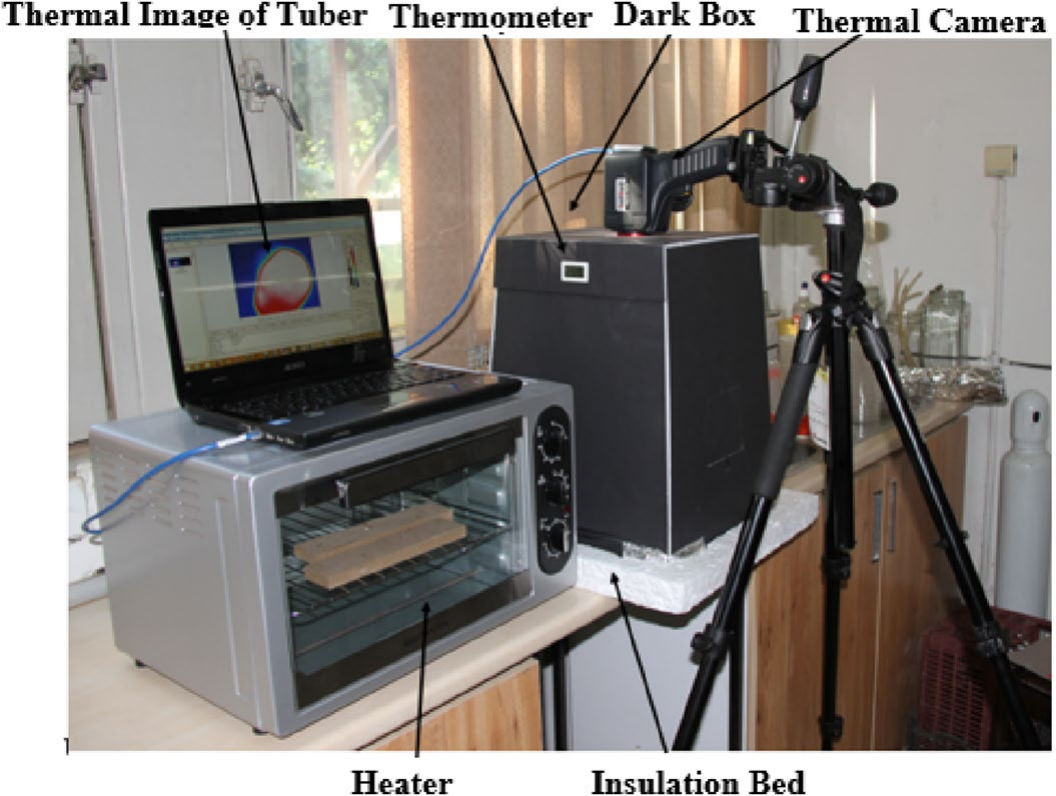
The experimental bed and thermography system^42^.

There is a need to prepare an experimental bed for diagnosing dry rot disease in potatoes. Figure 3 shows the experimental bed and thermography system used in this study. This system consisted of a thermal camera, a dark box, a thermometer, a computer, communication cables, a fan, a heater, and an insulated bed. The thermographs were taken by an infrared thermographic camera (G120 model, NEC Avio, Japan). The specifications of this camera are as follows, resolution, 240 × 320 pixel, the ability to receive a spectral range of 8–14 µm, thermal sensitivity, 0.04 °C at 30 °C, and spectral resolution (field of view), 1.78 mrad. It is noteworthy that the preparation of potato thermographs requires the estimation of their emissivity.

Since thermography cameras receive the emission and reflection of infrared waves of objects and there are the reflections of many other objects in the environment, the above-mentioned system contained a black box to counteract the effect of extra reflections. This box is an insulated chamber whose internal temperature is controllable; if the internal temperature exceeds 22 °C (room temperature), the fan begins operating to reduce the temperature to 22 °C. In addition, active thermography using a box heater (oven) was employed to make a noticeable thermal difference between healthy and dry rotted tubers.

In the present research, the applied active thermography treatments included two levels of heating temperature (60 and 90 °C) and four levels of natural cooling time (10, 25, 40, and 70 s). For active thermography, 60 and 90 °C were considered for heating potato tubers by an oven for 60 s. As the heating process is conducted in a short time and temperature rising in very low, the influence of the surface temperature of potato tubers on the total emissivity is negligible^34^.

The potato tubers were put on a wooden pan, individually, then tubers were transported from the oven into the dark box after heating to capture the thermal images after 10, 25, 40, and 70 s after natural cooling in room temperature. The clothe gloves were used to avoid heat transfer from the tubers to the hands and vice versa.

### Assessment of disease severity

The severity of potato dry rot was measured based on the Wiersema Criteria^35^. In the criteria, a scoring subset of 0 to 3 or A to D grade is used to classify the severity of the disease. The interval between disease development and complete destruction may varies depending on the type of inoculated fungus. It took three weeks for the severity of the disease to reach Grade 3 or D in a dark environment at 25 °C. Therefore, the effects of potato dry rot were evaluated weekly.

The development of potato dry rot in the contaminated tubers was classified under four classes as follows, healthy tuber, first-degree destruction, second-degree destruction, and third-degree destruction.

### Statistical analysis

**Figure 4:**
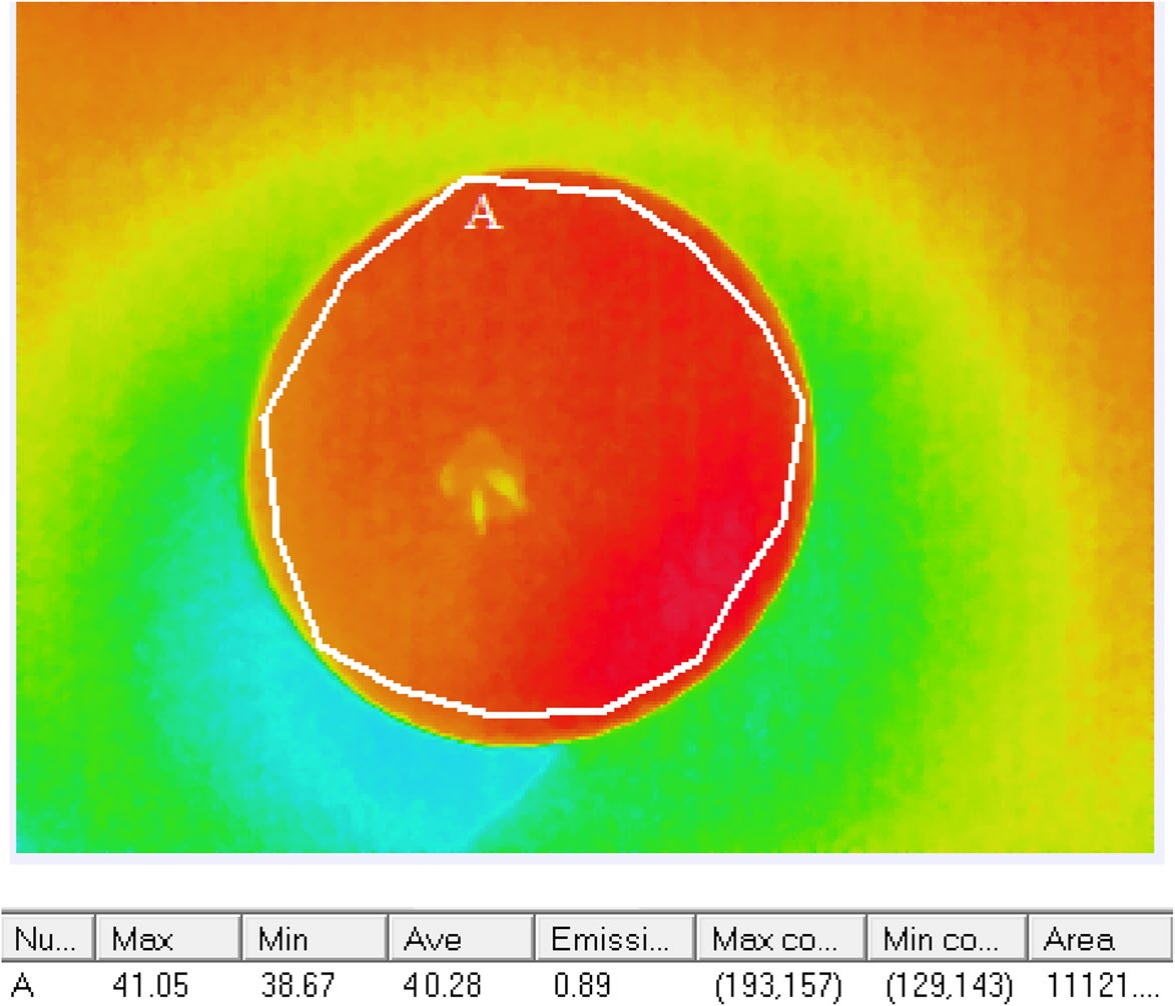
Determination of the surface temperature of the tubers by the InfReC Analyzer NS9500 Standard (NS9500).

To investigate the disease development level in the surface of the tubers, the temperature of the tuber surfaces was measured by InfReC Analyzer Software (Analyzer NS9500 Standard) (Fig. 4). The InfReC Analyzer is a high-performance software which enables real-time measurement, analysis, and report generating from thermal image and visible image. After measuring the temperature of the healthy and infected potato samples in different disease levels, the temperatures were analyzed to find the best heating treatment. The best treatment is a heating process in which the highest thermal difference between healthy and dry rotted tubers in different severity levels is obtained. To this end, a factorial experiment based on the randomized complete block design was conducted with three replications. The considered factors were oven temperature (60 and 90 °C), cooling time (10, 25, 40, and 70 s), and tuber destruction degree (at four level). The surface temperature of potato tubers was assessed by variance analysis and mean comparison was done by Duncan’s multiple range test method using SPSS-26 (2019) Software.

### Thermal image processing

**Figure 5:**
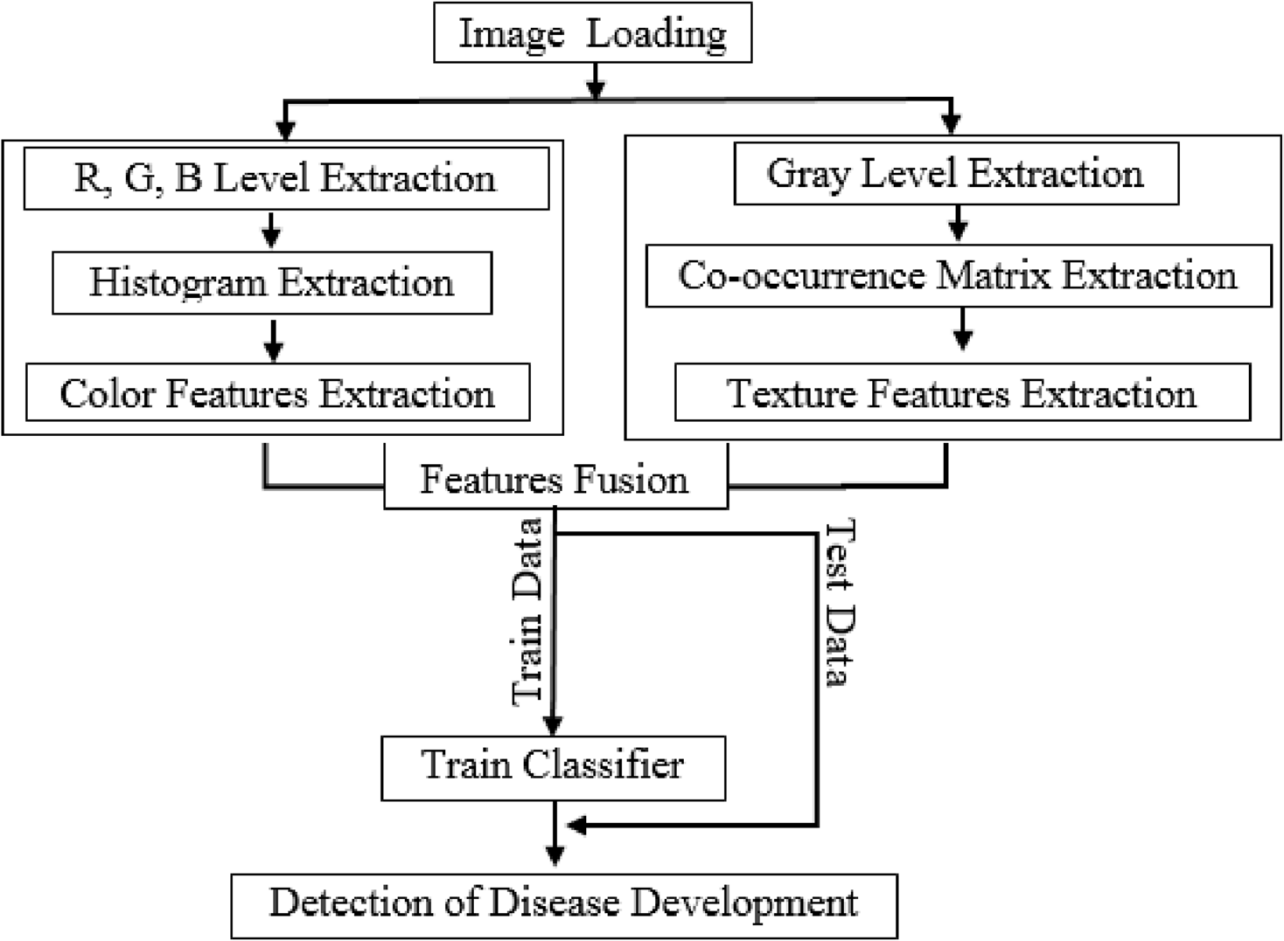
Thermal image processing to specify disease in potato tubers.

The image processing of the acquired thermal images was done to detect the healthy and infected tubers at different diseases levels. Figure 5 depicted the conducted steps in this regard.

### Tuber differentiation by threshold method

**Figure 6:**
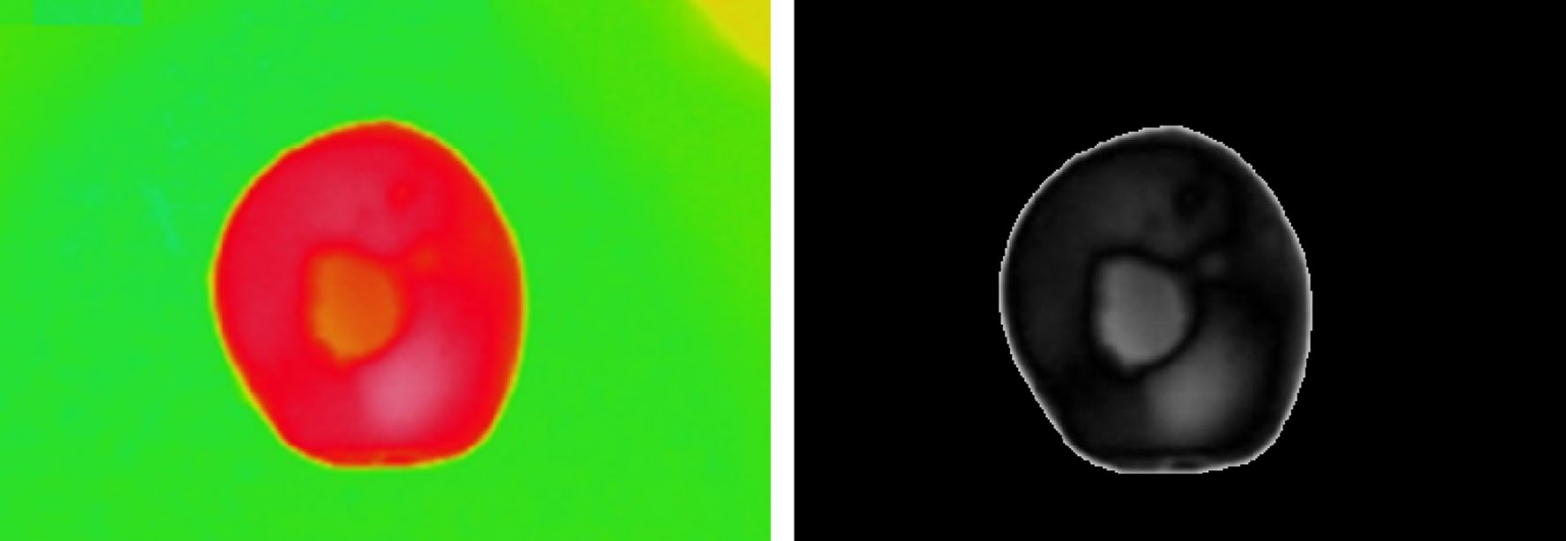
Applying threshold method on thermal images (left) to differentiate the diseases (right) of potato tubers.

Threshold is one of the most convenient ways to find specific areas in an image. Threshold is applied to grayscale images to obtain a binary image in which the objects are precisely differentiated from each other^36^. Otsu’s method is an image processing method that separates pixels into two classes, foreground and background (light and dark areas). Applying Otsu’s algorithm on an image as Fig. 6-left, cause to have an output as Fig. 6-right. Differentiation of tuber areas on thermographs is the first step in image processing based on Otsu’s method.

### Feature extraction

It was necessary to compare the thermographs in order to differentiate dry rotted potato tubers from healthy ones. To do this, the color and texture features of each thermograph were extracted. To extract color features, the red, green, and blue histograms were extracted from each thermograph and then six first-order statistical features (i.e. mean, standard deviation, mean square root, variance, skewness, and kurtosis) were extracted from the surface of the histograms. Finally, 18 color features were extracted from each thermograph.

Texture analysis has been widely used for crop quality assessment in recent decades, i.e. classification and detection of damaged areas of crops^37^. An important way to describe images is texture quantification. Although there is no formal definition for texture, this descriptor intuitively provides criteria for features such as smoothness, coarseness, and regularity^38^. The co-occurrence matrix is used for the extraction of location-based texture features, which is highly applied in texture analysis^39^. In this study, second-order statistical features (e.g. correlation, contrast, homogeneity, energy, and entropy) were extracted from the co-occurrence matrix at four angles of 0, 45, 90, and 135° with a distance of one of the thermographs, accounting for 20 texture features from each thermograph. See reference No. 32 for more information on the features extracted from the co-occurrence matrix. Each thermograph was described by 38 statistical features, which were used to develop the models for predicting the severity of tuber texture destruction.

### Classification

Artificial neural networks (ANN) method has been widely applied for the non-destructive evaluation of crops and food products over the past decade^9,40–43^, In the present study, this method was used to classify the severity of dry rot in potato tubers. The ANN method is a dynamic system to derive the knowledge from the input–output data for prediction and classification tasks^12^. The multilayer perceptron (MLP) model was considered in the ANN constructor. In the model included 38 neurons in the input layer (the number of the features) and four neurons in the output layer. The number of the neurons in hidden layer have been changed to reach a model with higher classification accuracy, so that 10 neurons was obtained as the number of the hidden layer of the best model. Seventy percent of the data was used for training and the remained was used for test the classifier model. Also support vector machine (SVM) was applied for classification of the different potato groups. The method was used due to achieve better results with fewer data^31^. The accuracy of SVM classifier models were evaluated for classification of different products^44,45^. The correct classification rates of the used methods were considered as model accuracy to compare their ability in identifying the progress level of the disease.

## Data Availability

The datasets used during the current study are available from the corresponding author on reasonable request.
